# Integrated and Comprehensive Diagnostics: An Emerging Paradigm in Precision Oncology

**DOI:** 10.3390/cancers18020327

**Published:** 2026-01-21

**Authors:** Kakoli Das, Jens Samol, Irfan Sagir Khan, Bernard Ho, Khoon Leong Chuah

**Affiliations:** 1 Department of Pathology, Tan Tock Seng Hospital, Singapore 308433, Singapore; 2 Department of Medical Oncology, Tan Tock Seng Hospital, Singapore 308433, Singapore; 3 Lee Kong Chian School of Medicine, Nanyang Technological University, Singapore 639798, Singapore; 4 Department of Medical Oncology, Johns Hopkins University, Baltimore, MD 212198, USA

**Keywords:** -omics, genomics, transcriptomics, proteomics, precision oncology, molecular pathology

## Abstract

Recent advances in molecular pathology, driven by integrated and comprehensive diagnostic approaches, have significantly advanced precision oncology. By leveraging multiomics technologies, molecular pathology enables the simultaneous assessment of genomic alterations, transcriptomic profiles, proteomic activity, and metabolic states integrated with conventional pathological evaluation to better explain tumour biology and behaviour. Large-scale international consortia, including The Cancer Genome Atlas (TCGA) and the Clinical Proteomic Tumour Analysis Consortium (CPTAC) have systematically demonstrated the value of harmonised multiomics analyses in defining tumour subtypes, uncovering functional dependencies, and generating clinically actionable insights. Evidence from coordinated precision oncology initiatives, such as the National Cancer Institute—Molecular Analysis for Therapy Choice (NCI-MATCH) trial further indicates that treatment strategies guided by molecular pathology profiling are associated with improved clinical outcomes, including progression-free survival in molecularly selected patient populations. Consequently, molecularly stratified treatment approaches are increasingly required in routine clinical practice to enable targeted therapies for selected tumour entities. Integration of molecular data with functional and clinical outcomes has further facilitated the detection of emerging mechanisms of therapeutic resistance and heterogeneous treatment responses. Importantly, studies have shown that reliance on genomic analysis alone is insufficient to achieve optimal targeted therapy, underscoring the need for multi-layered molecular interrogation. This review highlights the biological and clinical relevance of multiomics integration, emphasising its critical role in comprehensive morpho-molecular tumour assessment and functional analyses while providing clinicians with a practical framework for interpreting integrated molecular diagnostics and addressing the methodological and translational challenges that must be overcome to enable broader implementation of precision oncology in routine practice.

## Simple Summary

Cancer care is becoming more personalised due to advances in molecular testing. In the past, tumours were diagnosed mainly by examining tissue under a microscope. Today, new technologies allow clinicians to study cancer in detail by analysing genes, gene activity, proteins, and the biological processes that support cancer cell survival. When this molecular information is combined with routine pathology, it provides a clearer understanding of why tumours behave differently and why patients respond differently to treatment. Large research studies have shown that this integrated approach can help clinicians better classify cancers, choose more effective therapies, and understand why some treatments stop working overtime. Although challenges remain in standardising tests and interpreting complex data, integrated diagnostic approaches represent an important step toward more precise and personalised cancer care. This review highlights the role of combining multiple molecular tests in transforming cancer diagnosis and treatment with the aim of improving patient outcomes.

## 1. Introduction

Cancer is a leading cause of death worldwide, with nearly 20 million new cases and almost 10 million deaths reported in 2022. The most common cancers globally include lung, breast, colorectal, and prostate malignancies [1]. Patient outcomes are generally poor, primarily due to delayed diagnosis and limited efficacy of existing therapeutic approaches. Furthermore, the extensive molecular and clinical heterogeneity across cancer types poses a major challenge to management, thereby underscoring the critical need for precision medicine strategies tailored to individual profiles.

Pathology has played a pivotal role in diagnosis and in decision making for patients’ treatment. It has evolved into a multidisciplinary field, encompassing histopathology, cytopathology, molecular pathology, and whole slide imaging technology or digital pathology, each contributing uniquely to diagnostics and patient care. Molecular pathology, which is a combination of molecular biology, genetics, and pathology, considers the molecular alterations occurring in response to environmental and other intrinsic factors that drive disease processes and elucidate the key pathways that contribute to the disease.

Historically, conventional stains such as haematoxylin and eosin, and the introduction of immunohistochemistry (IHC) in the 1960s, revolutionised diagnostic pathology by complementing tissue morphology with protein expression profiles [2,3]. The advent of fluorescence in situ hybridisation (FISH) almost two decades later enabled the detection of specific cytogenetic abnormalities and copy number variations, thereby extending the diagnostic framework and providing insights beyond those attainable with IHC alone [3,4]. However, to achieve a more comprehensive understanding of a patient’s tumour, it became essential to uncover the molecular underpinnings of the disease. [5]. Recent advances in single-gene PCR-based assays [6] for detecting single gene alterations such as *EGFR* in lung cancer have also enabled a more comprehensive understanding of cancer type, uncovering alterations that disrupt cellular homeostasis and drive malignant transformation. However, these are often insufficient when tumours are poorly differentiated, metastatic, of unknown origin, or show therapy-induced molecular drift, as cancers rarely rely on single driver mutations [7,8,9]. The combined effect of co-occurring events determines the biological aggressiveness of the tumour and its response to therapy and eventual clinical outcome, which can be achieved only using high-throughput multiomics technologies.

Multiomics technologies have significantly expanded the resolution and scale of molecular analyses, including genomics, transcriptomics, epigenomics, proteomics, metabolomics, to provide a comprehensive understanding of biological systems, identification of novel biomarkers, elucidation of disease mechanisms, and stratification of patients for precision therapies. While genomic profiling identifies the mutational and structural variants that initiate oncogenesis [10], transcriptomic analyses capture the downstream consequences of these alterations on gene expression and pathway activation [7]. Likewise, epigenomic mapping reveals the DNA methylation, histone modifications, and chromatin organisation that shape transcriptional programmes and cellular plasticity. Proteomic and metabolomic approaches complement these alterations by quantifying protein abundance, post-translational modifications, and metabolic fluxes, thereby capturing cellular function in real time [11,12,13].

**Figure 1:**
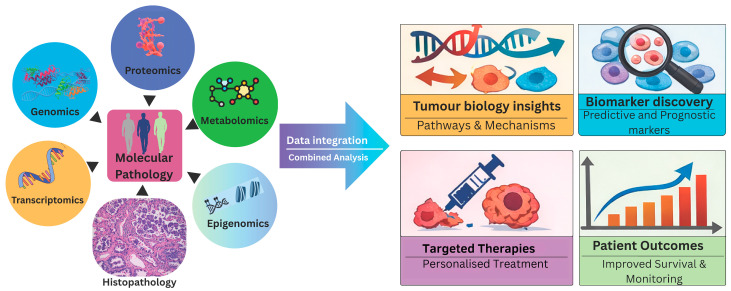
Significance of integrated multiomics analysis for precision oncology. Schematic overview of an integrated molecular pathology framework illustrating the convergence of histopathology with multiomics profiling showing the biological and clinical significance for tailored and effective cancer care.

Large multiomics consortia have used such integrative analyses to detect tumour subtypes that are more biologically and clinically informative than those discernible by any single platform alone. Each molecular platform captures different biological processes such as drivers, expression, regulation, and microenvironment [14,15,16]. Therefore, understanding these complex molecular interactions is crucial for accurate disease characterisation, prognostication, and the development of targeted therapeutic strategies. In this review, we highlight the biological and clinical significance of integrated multiomics approaches, focusing on their comprehensive molecular insights that have improved our understanding of tumour biology, patient care, and informed clinical management (Figure 1).

This review was conducted as a focused review of the literature on integrated multiomics approaches in precision oncology. Relevant studies were identified primarily through searches of PubMed using combinations of keywords including multiomics, precision oncology, molecular pathology, genomics, transcriptomics, proteomics, and metabolomics. Priority was given to high-quality primary studies, large international consortia, clinical trials, and reviews published in peer-reviewed journals.

## 2. Biological Significance of Multiomics Integration

The biological significance of multiomics integration lies in its ability to capture the functional complexity of cancer beyond single-omic analysis. Integrating the analysis from genetic alterations to downstream regulatory, signalling, and metabolic consequences results in a system-level understanding and more accurate mapping of genotype to phenotype. The integrative framework reveals biological heterogeneity among tumours sharing similar driver mutations, uncovers epigenetic and post-translational mechanisms that modulate pathway activity, and elucidates dynamic interactions between cancer cells and the tumour microenvironment.

### 2.1. System-Level Understanding

Multiomics technologies have transformed cancer research from a gene-centric focus to a systems-level framework, enabling comprehensive exploration of the molecular networks that regulate tumour behaviour. A study that applied an integrative molecular clustering approach combined data on chromosome-arm aneuploidy, DNA methylation, transcriptomic expression, and proteomic profiles across thousands of tumour samples and found that the resulting tumour clusters were strongly associated with histology, tissue type, and anatomic origin rather than restricted to single tumour types or isolated gene mutations [16]. This analysis demonstrated that patterns of genomic instability, epigenetic regulation, and downstream gene and protein expression collectively reflected cell-of-origin constraints and tissue-specific biology, providing a systems-level classification framework that transcended traditional histopathological categories. Such stratification could improve clinical trial design by enabling inclusion criteria and subgroup analyses that account not only for mutations and oncogenic pathways but also for the broader anatomic, histological, and cell-of-origin contexts that influence tumour behaviour and therapeutic response.

Complementing this, Integrative Clusters (IntClust) classification of breast cancer showed systems-level analysis using multiomics data that refined tumour taxonomy beyond traditional histopathological and single-gene approaches. Derived from the integration of copy number alterations, gene expression profiles, and genomic instability patterns, the IntClust framework defined ten biologically distinct subtypes (IntClust 1–10), each characterised by specific genomic drivers, transcriptional programs, and clinical behaviour. At a systems level, IntClust subtypes captured the interplay between structural genomic alterations and downstream functional consequences, associating chromosomal instability, oncogenic pathway activation, and tumour phenotype. For example, IntClust 1 and 2 were driven by 17q23 and 11q13 amplifications, respectively, implicating cell-cycle and growth factor signalling networks, while IntClust 5 was defined by *ERBB2* amplification and *HER2*-driven signalling. In contrast, IntClust 3 and 4 comprised genomically stable, oestrogen receptor-positive tumours in which limited structural disruption restricted transcriptional diversity, corresponding to more indolent clinical behaviour and favourable outcomes [17].

A comprehensive multiomics cohort of 773 Chinese patients with breast cancer, known as the Chinese Breast Cancer Genome Atlas (CBCGA), was established and systematically analysed across genomic, transcriptomic, proteomic, metabolomic, radiomic, and digital pathology modalities. Compared with breast cancers in predominantly White cohorts, the Chinese cohort showed ancestry-specific molecular features, including a higher proportion of *HER2*-enriched tumours and more frequent *ERBB2* amplification, as well as distinct mutation patterns such as increased targetable *AKT1* mutations. Integrated multiomics analysis also identified potential therapeutic vulnerabilities and enabled stratification of patients into groups with differing recurrence risks, providing new insights into breast cancer biology and ancestry specificity relevant for precision treatment approaches [18].

System-level molecular profiling (ion channel expression), including assessment of key regulatory proteins such as TRPM2 in brain tumours (glioma), has provided insight into functional pathways that influence tumour behaviour and therapeutic response [19]. Multi-platform untargeted metabolomic analyses, integrating liquid chromatography quadrupole time-of-flight mass spectrometry (LC-QTOF-MS), gas chromatography quadrupole time-of-flight mass spectrometry (GC-QTOF-MS), and targeted amino acid profiling, were applied to characterise metabolic alterations associated with breast cancer molecular subtypes and disease progression [20]. The study demonstrated the capacity of metabolomics to capture functional metabolic reprogramming, supporting its potential utility in early diagnosis, disease monitoring, and refined molecular characterisation.

Together, these findings suggest that multiomics integration offers a more comprehensive, system-level understanding of tumour biology that cannot be captured by any single technology or platform alone.

### 2.2. Mapping Genotype with Phenotype

A multiomics approach integrating genomics with proteomics revealed phenotypic effects. The TCGA Network highlighted genotype–phenotype relationships through multi-platform analyses, demonstrating that DNA sequencing alone fails to capture many tumours with phosphoproteomic evidence of RTK/RAS/RAF and PI3K pathway activation. Identical genotypes could yield divergent phenotypes, suggesting the presence of additional, yet uncharacterized mechanisms governing pathway activation [21].

The CPTAC subsequently addressed this hypothesis, demonstrating that integration of genomics with proteomics provided a critical functional dimension that advanced translational cancer biology. By applying standardised proteomic workflows to the TCGA-characterised colorectal, ovarian, and breast cancers, CPTAC revealed clinically actionable targets that could be detected using routine clinical testing [22]. In the CPTAC colorectal cancer study [23], RNA levels failed to reliably predict protein abundance, and most focal amplifications did not translate into proportional protein increases. Five proteomic subtypes emerged, including one associated with highly aggressive behaviour. The study also identified candidate therapeutic targets detectable only through integrated analyses. In the breast cancer study [24], proteogenomic analysis uncovered new markers and signalling pathways in tumours carrying *PIK3CA* and *TP53* mutations. Ten genes with copy number alterations were functionally linked to corresponding trans-protein effects. Notably, the frequent 5q deletion in basal-like tumours implicated *SKP1* and *CETN3* as regulators of *EGFR* and *SRC* expression. Phosphoproteomic clustering also revealed a GPCR subgroup, a subset of tumours defined by activation of G-protein-coupled receptor signalling at the protein/phosphoprotein level rather than by distinct genomic or transcriptomic features.

Similar proteogenomic profiling was also carried out in lung adenocarcinoma [15,25,26] and clear cell renal cell carcinoma [27]. The lung adenocarcinoma analysis revealed frequent activation of the phosphatase PTPN11 (SHP2) in *EGFR*, *ALK*, and *KRAS*-driven tumours, nominating it as a cross-genotype therapeutic target. Moreover, *KRAS* activation in *KRAS*-mutant tumours induced PD-L1 expression via the MEK-ERK-ETV4 axis, mapping a genomic driver to an immune regulatory phenotype [25]. Additional integrative analyses identified three reproducible *KRAS*-mutant subgroups defined by co-mutations in *STK11*/*LKB1*, *TP53*, or *CDKN2A/B* and by differential NKX2-1 expression, each with distinct immune landscapes and therapeutic targets [26,28]. In treatment-naive clear cell renal cell carcinoma (ccRCC), proteogenomic profiling identified dysregulated proteins across cellular processes influenced by genomic alterations, including oxidative phosphorylation-related metabolism, protein translation, and phospho-signalling pathways, providing a biological rationale for informed treatment selection based on ccRCC pathobiology [27].

A key insight from these studies was that transcript abundance alone was often insufficient to predict protein levels, and that post-translational modifications, particularly phosphorylation events, were essential indicators of pathway activation and cellular phenotype, which could be captured by multiomics integration. TCGA established foundational genomic, epigenomic, and transcriptomic landscapes across tumour types but was largely limited by its retrospective design and variable clinical annotation. In contrast, CPTAC extended these frameworks by incorporating quantitative proteomics and phosphoproteomics, enabling closer linkage between genomic alterations and functional pathway activity, although cohort sizes and tumour-type coverage remained more restricted. Overall, these major international consortia have been instrumental in advancing integrated molecular characterisation of cancer. However, differences in methodological scope, analytical depth, and clinical applicability highlight their complementary strengths and emphasise the need for harmonised, clinically oriented multiomics frameworks.

### 2.3. Tumour Heterogeneity

Tumours sharing the same initial “trunk” driver mutation can display striking heterogeneity, shaped by co-existing sub clonal genetic and epigenetic divergence as well as differences in the tumour microenvironment. As tumours expand, ongoing genomic instability drives the continual accumulation of both driver and passenger mutations in different cell populations, giving rise to distinct subclones with unique mutational profiles, a phenomenon collectively referred to as intra-tumoural heterogeneity. Consistent with this concept, integrative analyses incorporating exome sequencing, chromosomal aberration assessment, and ploidy profiling across multiple spatially separated regions of renal carcinomas and their matched metastatic sites revealed a branched pattern of tumour evolution. This evolutionary architecture was marked by many somatic mutations that were confined to specific tumour regions rather than being uniformly present across the entire tumour mass [29]. Extending this paradigm to lung cancer, *EGFR*-mutant non-small cell lung cancer (NSCLC) exhibited pronounced metabolic heterogeneity, characterised by altered glucose metabolism and growth factor-driven pathways [30]. These observations highlighted that even tumours defined by a common oncogenic driver could adopt divergent metabolic and immune phenotypes, reinforcing the need for integrative, multiomic approaches to fully capture functional heterogeneity and informed precision therapeutic strategies.

Epigenetic heterogeneity describing variability in epigenetic modifications including DNA methylation, histone modification, and non-coding RNA expression also significantly impacted phenotypic changes. Among these, epithelial-to-mesenchymal transitions such as *ZEB1*, *SNAIL*, and *SLUG* conferred drug resistance to platinum-based chemotherapy in breast, ovarian, colon, and pancreatic cancer, providing a selection advantage to this epigenetic state [31]. A similar pattern emerged in the tumour microenvironment and immune escape mechanisms, significantly influencing prognosis and treatment response. The tumour microenvironment includes stromal cells, such as fibroblasts, mesenchymal stem cells, endothelial cells, and immune cells. Multiomics analyses identified ‘hot’ and ‘cold’ immune microenvironments, with a high degree of tumour infiltration in hot and almost no immune activity in the cold immune microenvironment [32]. This tumour microenvironment heterogeneity resulted in substantial inter-individual variability in tumour characteristics. Building on this work, a large multiomics dataset of triple-negative breast cancer (TNBC) was subsequently generated, which further revealed distinct tumour microenvironmental characteristics across TNBC tumours. The tumour phenotypes were classified into immune dessert clusters with low microenvironment cell infiltration; innate immune inactivated clusters with resting innate immune cells and non-immune stromal cell infiltration; and immune inflamed clusters with abundant adaptive and innate immune cell infiltration. These phenotypes correlated with prognostic efficacy, suggesting significant progress towards personalised immunotherapy strategies for patients with TNBC [33].

Therefore, multiomics technologies demonstrated the complexity of tumour heterogeneity and tumour–microenvironment interactions at both the molecular and cellular levels, providing an integrated view of how intrinsic tumour features and extrinsic signals collectively shape cancer behaviour.

### 2.4. Regulatory Mechanisms

Besides tumour heterogeneity, multiomics approaches have also revealed the critical role of methylation-directed regulatory networks in cancer. Epigenomic profiling has shown that promoter hypermethylation can silence tumour suppressor genes even in the absence of pathogenic mutations, effectively mimicking loss-of-function events and driving malignant progression, thereby highlighting epigenetic dysregulation as an independent and potent determinant of cancer phenotypes [34]. Integrated epigenomic–transcriptomic analyses of lung cancer identified novel methylation driver genes with consistent, expression-linked effects, underscoring their diagnostic and therapeutic relevance [35]. Likewise, in breast cancer, multiomics studies highlighted gene regulatory mechanisms that shaped tumour phenotype, prognosis, and therapeutic response. For instance, the *CT83* gene is frequently activated in TNBC but silent in non-TNBC, normal adult non-testis tissues, and blood cells, which is associated with poorer overall survival, representing a promising target for cancer immunotherapy and the development of new diagnostic and prognostic biomarkers [36]. Similarly, *CBX2* and *CBX7* exhibited antagonistic roles in metabolic reprogramming, regulating glucose metabolism and predicting both patient outcome and drug sensitivity [37]. These findings emphasise that in many tumours, epigenetic alterations exert a stronger influence on gene expression than DNA sequence changes, with methylation-mediated silencing events often essential for cancer cell survival.

A study interrogating methylation-associated regulatory variation across 125 pan-cancer and glioblastoma (GBM) driver genes together with 52 reference genes demonstrated that DNA methylation modulated the activity and mode of action of gene-associated silencers and enhancers in both controlled systems and intact cancer genomes. High-resolution mapping of regulatory methylation sites further revealed that *cis*-regulatory domains were organised into overlapping, gene-specific regulatory networks composed of multiple positive and negative regulatory units. Notably, variation in a limited number of key methylation sites within these units was sufficient to explain inter-patient differences in cancer gene expression among GBM cases, highlighting the central role of methylation-directed regulatory architecture in shaping tumour phenotypes [38]. Thus, multiomics analyses showed how epigenetic mechanisms orchestrated regulatory networks and tumour behaviour, offering opportunities for diagnostic, prognostic, and therapeutic applications.

## 3. Clinical Significance of Multiomics Integration

Integration of multiomics technologies has enhanced clinical interpretation of cancer by refining the evidence framework used to classify tumour alterations. For Tier I alterations, which have strong therapeutic or prognostic significance, multiomics concordance has reinforced their clinical actionability and supported guideline-based management as defined by AMP/ASCO/CAP. For Tier II alterations, which include co-mutations supported by emerging or lower-level evidence, multiomics data has demonstrated consistent functional effects, such as altered gene expression, pathway activation, or epigenetic dysregulation. Tier III alterations, including the variants of uncertain significance (VUS), have become more interpretable when evaluated through multiomics data. Transcriptomic or methylation signatures have shown the functional impact of these variants, their potential pathway involvement, or possible germline predispositions [39,40]. Multiomics integration informs multiple clinical avenues, including integrative subtyping, therapeutic stratification, identification of biomarker-driven targets, precision clinical trial design, and enhanced longitudinal disease monitoring. Together, these approaches have strengthened clinical decision-making by enabling more precise tumour subtype definition and generating actionable insights for prognosis, therapeutic selection, and disease monitoring.

### 3.1. Integrative Subtyping

Integrative classification has refined prognostication beyond conventional staging or mutation-based approaches. One such example is glioblastoma, where integrative analyses identified *IDH1/2*-mutant gliomas with a CpG island methylator phenotype (G-CIMP) that correlated with improved survival and differential treatment response, leading to distinct clinical management guidelines [41]. In endometrial carcinoma, TCGA multiomics classification defined four molecular subgroups: (a) *POLE* ultramutated with very high tumour mutational burden (exceeding 100 mut/Mb), (b) MSI-high, (c) copy-number low, and (d) copy-number high, each with distinct prognosis and therapy sensitivities. This molecular taxonomy has since been incorporated into clinical practice and treatment algorithms [42,43]. A similar iCluster analysis of uveal melanoma identified two major integrative subtypes—M3 and D3—each defined by distinct multiomics landscapes. The M3 subtype was associated with significantly worse overall survival than D3, providing a comprehensive framework for classifying uveal melanoma into high- and low-risk groups for metastasis [44]. Similar studies in melanoma demonstrated integrative cancer subtypes (CS)—CS1 and CS2—with clear prognostic differences. The CS1 subtype showed favourable survival outcomes compared to CS2, which demonstrated poorer overall survival and was predicted to be less responsive to immunotherapy [45].

Molecular integrative analysis also led to the identification of four consensus subtypes in colorectal cancer by an international Colorectal Cancer Subtyping Consortium with clear biological interpretability and subtype-based targeted interventions. The Consensus Molecular Subtypes (CMS) included four subtypes (CMS1–CMS4): (a) CMS1—the Microsatellite Instability Immune subtype with high MSI, strong immune activation, and frequent *BRAF* mutations often associated with better prognosis in early-stage disease but poorer response to standard chemotherapy in advanced stages; (b) CMS2—the canonical subtype with chromosomal instability, wingless related integration site (WNT) and *MYC* proto-oncogene pathway activation, epithelial differentiation, and better response to standard therapies; (c) CMS3—the metabolic subtype, featuring an epithelial subtype with metabolic dysregulation and frequent *KRAS* mutations with a distinct metabolic program and potential for metabolism targeted therapies; and (d) CMS4—the mesenchymal subtype with prominent TGF-β activation, stromal invasion, angiogenesis, and epithelial-to-mesenchymal transition (EMT) diagnosed at more advanced stages (III and IV) with worse overall survival and relapse-free survival, more resistant to standard therapies [46].

Advanced computational frameworks, including network-based and graph theoretical integration methods, enable multiomics data to be interpreted within biologically and clinically meaningful contexts. A fusion network-based approach (FUNMarker) was developed to identify prognostic biomarkers in a breast cancer study by integrating gene expression data with multiple layers of biological information. Patient samples were first clustered based on gene expression patterns to account for inter-tumour heterogeneity, after which genes were evaluated according to biological function, prognostic relevance, and association with known disease genes. FUNMarker identified biologically interpretable biomarkers with an improved ability to distinguish patient subgroups with different prognostic outcomes, illustrating the value of network-based multiomic integration for refined risk stratification in breast cancer [47].

Together, these findings highlight the strong clinical impact of multiomics technologies, demonstrating the role of integrative subtyping in prognostic assessment and guiding personalised therapy.

### 3.2. Biomarker-Driven Drug Targets

Multiomics frameworks can uncover functional dependencies and candidate drug targets by linking genomic alterations to their transcriptional and proteomic consequences. For instance, *EGFR*-mutant NSCLC frequently displays dependency on aberrant *EGFR* signalling for tumour maintenance, explaining the clinical efficacy of *EGFR* tyrosine kinase inhibitors (TKIs) [48]. However, *EGFR* mutation alone is not universally sufficient for oncogenic transformation, and co-occurring genomic, epigenetic, and pathway-level alterations can modulate therapeutic sensitivity and resistance. Moreover, *EGFR* mutations differ in their structural and functional properties, and classification based on structure–function relationships rather than exon location alone improves prediction of TKI response [49]. Beyond *EGFR* itself, multiomics and functional studies have revealed additional co-dependencies, such as concurrent *EGFR* mutation and *MET* amplification, which predict benefit from combined *EGFR* and *MET* inhibition [50]. Similarly, integrated immune transcriptomic and methylation profiling identifies immune-inflamed tumour subtypes associated with improved responsiveness to immune checkpoint blockade [51]. Collectively, these biomarker-driven dependencies provide a rationale for precision combination therapies and biomarker-enriched clinical trial designs. The LIQUIK-01 study has clearly demonstrated that using circulating tumour DNA (ctDNA) and circulating tumour RNA (ctRNA)-based platforms, as well as combining tissue and liquid biopsies, increases the detection of targetable alterations [52].

Multiomics profiling has uncovered a broad spectrum of other biomarker-defined therapeutic dependencies in NSCLC, many of which are now clinically actionable. Prominent examples include oncogenic rearrangements involving *ALK* [53], *ROS1* [54], *RET* [55], and *NTRK* [56], which confer sensitivity to kinase inhibitors, as well as *BRAF* V600E [57] or *KRAS* G12C [26] mutations that are effectively targeted through MAPK pathway inhibition. Additional multiomics-informed biomarkers linked to targeted treatment strategies include *HER2* mutations amenable to antibody–drug conjugates such as trastuzumab deruxtecan [58], PD-L1 expression [59], and high tumour mutational burden (TMB) [60], which predict benefit from immune checkpoint blockade and alterations in DNA damage repair pathways that confer vulnerability to PARP inhibitors [61].

Beyond these established biomarkers, multiomics studies have identified several additional therapeutic dependencies in other cancer types such as breast cancer, where *PIK3CA* mutations and *BRCA1/2*-associated homologous recombination deficiency predict response to PI3K and PARP inhibitors, respectively [62,63]. In prostate cancer, AR pathway alterations and DNA repair gene defects define sensitivity to next-generation AR inhibitors and PARP inhibitors [64]. Melanoma studies demonstrated that *BRAF* V600 mutations and immune gene expression signatures guided targeted and immunotherapy selection [65], while pancreatic cancer analyses highlighted *KRAS*-driven metabolic dependencies as actionable vulnerabilities [66]. Overall, these biomarkers have delineated tumour-specific oncogenic and immune dependencies and guided the rational selection of highly effective therapies.

### 3.3. Therapeutic Stratification

Building on these findings, integrative molecular profiling enabled therapeutic stratification by grouping patients into biologically defined subtypes with distinct pathway dependencies and variable drug sensitivities (Table 1).

A canonical example of therapeutic stratification enabled by integrative genomics is the identification of four biologically distinct clusters in diffuse large B-cell lymphoma, with each subtype predicting sensitivity to specific targeted agents such as Bruton’s tyrosine kinase and BCL2 inhibitor. These clusters comprise MCD (based on the co-occurrence of *MYD88*^L265P^ and *CD79B* mutations), BN2 (based on *BCL6* fusions and *NOTCH2* mutations), N1 (based on *NOTCH1* mutations), and EZB (based on *EZH2* mutations and *BCL2* translocations) [67].

Similarly, another integrative analysis representing functional therapeutic stratification driven by pathway dependencies identified master kinases responsible for affecting phenotypic hallmarks of functional glioblastoma subtypes, Glial Progenitor-like Mesenchymal (GPM), Proneural Progenitor-like Receptor-tyrosine kinase-driven (PPR), and Metabolic/Terminally differentiated/Classical-like (MTC) with distinct kinase signalling and metabolic features. The subtypes differed in sensitivity to inhibitors targeting subtype-specific master kinases (e.g., PKCδ, DNA-PK), indicating possible targeted therapy strategies tailored to the subtype [68].

Therapeutic stratification with biomarker-guided drug prioritization identified six potential therapeutic drugs, including the MEK inhibitors, Selumetinib and Trametinib, and their combinations in a CA19-9-positive intrahepatic cholangiocarcinoma (ICC) clinical cohort study that exhibited poorer overall survival and relapse free survival. The integrated study encompassed clinical, genomic, transcriptomic, and immune landscapes, identifying metabolically distinct, glycolysis-enriched cellular populations, elucidating their tumour-promoting interactions, and providing new biological insights into ICC and personalised therapeutic approaches [69]. Similarly, proteogenomic profiling of 110 lung adenocarcinoma tumours and 101 matched normal tissues revealed molecular subgroups defined by driver mutations, patient origin, and gender, highlighting distinct pathway dependencies. Integration of proteomic and phosphoproteomic data identified downstream effects of *KRAS*, *EGFR*, and *ALK* alterations and uncovered potential therapeutic targets, sensitive to pathway-specific inhibitors. Immune profiling further distinguished subgroups with immunosuppressive features, such as *STK11*-associated immune-cold tumours. These findings demonstrated how multiomics analyses prioritised actionable targets, informed patient-specific therapy selection, and supported mechanism-driven precision oncology across tumour types [15].

In high-grade serous ovarian cancer, a major challenge is that many patients eventually develop resistance to platinum-based chemotherapy, which is the standard first-line treatment. A proteogenomic characterisation of chemo-refractory tumours identified a 64-protein signature predictive of platinum resistance. This signature not only allowed for stratification of patients likely to respond poorly to standard chemotherapy but also revealed therapeutic targets within the tumours, pointing to alternative targeted interventions. By integrating protein-level data with genomic and transcriptomic features, this approach demonstrated that functional molecular signatures could guide personalised therapy selection and prioritise drug candidates across patient cohorts and preclinical models, highlighting the translational potential of multiomics analyses for mechanism-driven precision oncology [70]. Integrative analyses have thus enabled the classification of patients into biologically distinct subtypes, each associated with specific therapeutic sensitivities, thereby guiding more precise and effective treatment strategies.

### 3.4. Precision Trial Design

In the context of precision oncology, innovative clinical trial designs have evolved to leverage integrative molecular profiling, enabling more efficient evaluation of targeted therapies and biomarker-guided treatment strategies. Traditional trials focused on single biomarkers are now being supplemented or replaced by master protocols, such as basket, umbrella, and platform trials, which facilitate simultaneous testing of multiple drugs and patient subgroups within a unified framework. Basket trials, like NCI-MATCH, enrol patients across different cancer types based on shared molecular alterations, allowing targeted therapies to be evaluated regardless of histology and identification of tumour-specific activity that may be obscured in conventional cohort studies [71].

Umbrella trials, such as Lung-MAP and I-SPY2, stratify patients within a single cancer type based on multiple biomarkers and assign them to matched therapeutic arms, improving the probability of demonstrating benefit in molecularly defined subsets [72]. Platform trials further build on this flexibility by dynamically adding or removing biomarker cohorts and treatment arms as new data emerge, enhancing adaptability and efficiency in evaluating complex therapeutic landscapes [73]. These designs often incorporate adaptive features, including interim stopping for lack of efficacy or selective enrichment of responsive patient subgroups, which can accelerate development timelines and focus resources on the most promising interventions [74]. They also assess endpoints such as progression-free survival, objective response rate, or molecular response, and they focus on molecularly selected patient populations [9]. Although outcome improvements have been reported in specific contexts, heterogeneity in study design, patient selection, and molecular testing platforms limits direct cross-study comparisons and highlights the need for harmonised trial designs and standardised evidence frameworks.

### 3.5. Disease Monitoring

Multiomics signatures have also transformed disease surveillance by enabling dynamic monitoring of tumour evolution. Integration of genomic alterations (e.g., emerging resistance mutations), transcriptomic reprogramming, and methylation or proteomic changes in ctDNA or exosomes have provided a non-invasive window into tumour behaviour over time. For example, serial liquid biopsy sequencing in *EGFR*-mutant NSCLC has revealed acquisition of *EGFR T790M* or *C797S* resistance mutations, accompanied by transcriptomic changes associated with epithelial–mesenchymal transition and drug tolerance [75]. In breast cancer, ctDNA and circulating tumour cells (CTCs) have been used to monitor treatment response, detect progression, and identify recurrence non-invasively, offering information on tumour dynamics that supplements traditional markers and imaging [76]. Plasma ctDNA has been shown to detect early molecular relapse in hepatocellular carcinoma patients after curative treatment, with ctDNA positivity correlating with shorter relapse-free survival, enabling earlier detection of recurrence compared with conventional methods [77]. CTC enumeration has prognostic value in metastatic prostate cancer; high CTC counts are independently associated with poorer progression-free and overall survival, illustrating that liquid biopsy markers could reflect disease status and guide monitoring.

Combining such multiomics liquid biopsy profiles enhances sensitivity for early relapse detection and offers insight into clonal selection under treatment pressure. This integrated monitoring allows clinicians to adapt therapy pre-emptively, before radiologic progression, thereby improving patient outcomes and minimising unnecessary exposure to ineffective regimens.

## 4. Conclusions

Clinical evaluation of cancer has evolved substantially over recent decades, driven by advances in multiomics technologies that enable high-resolution, multidimensional interrogation of tumour biology. Integration of molecular information across genomic, transcriptomic, proteomic, and metabolomic layers facilitates the identification of clinically relevant features and more precise refinement of disease subtypes in solid tumours. As a result, precision oncology is increasingly positioned to benefit from actionable insights generated through multiomics profiling to inform personalised strategies for cancer prevention, diagnosis, and treatment. Emerging theranostic platforms, including tumour microenvironment-responsive nanoparticles, also exemplify how detailed molecular and microenvironment profiling can inform personalised therapeutic strategies [78]. However, while research-driven precision oncology initiatives have clearly demonstrated the biological value of deep, multi-layered molecular characterisation, their scope and complexity often exceed what is currently feasible in routine clinical practice. In real-world settings, precision oncology continues to rely on clinically validated, cost-effective, and time-efficient assays, mostly focused on targeted genomics, immunohistochemistry, and selected transcriptomic or proteomic tests. Bridging the gap between discovery and practice will require further standardisation of assays, prospective clinical validation, and the development of integrated workflows tailored to routine pathology environments.

## 5. Future Developments

Despite its promise, clinical translation of multiomics data remains challenging. Molecular pathologists and clinicians must interpret increasingly complex datasets to determine clinical relevance and guide therapeutic decisions. Biomarkers integrated into standard-of-care practice should be supported by high-quality evidence from prospective trials demonstrating improved patient outcomes. While many predictive biomarkers have been identified, further validation is needed to optimise their clinical utility.

In immuno-oncology, tumour mutational burden (TMB) has emerged alongside PD-L1 expression and microsatellite instability as a potential predictive marker, with POLE/POLD potentially further stratifying non-MSI and TMB-low patient subsets [79]. However, TMB’s value as a standalone biomarker is limited and inconsistent across tumour types [80]. High TMB is associated with improved responses to immune checkpoint inhibitors in some cancers, but harmonisation of assessment protocols and evidence-based thresholds is required. Consequently, TMB is typically interpreted alongside other molecular and clinical factors, highlighting the need for integrated, multi-layered biomarker strategies [81].

Functional analyses and resistance monitoring provide insights into tumour evolution and therapeutic response but remain difficult to implement routinely. Targeted, clinically validated assays, including focused next-generation sequencing panels, immunohistochemistry, and liquid biopsy platforms, can be incorporated into existing workflows. Interpretation increasingly relies on molecular tumour boards, where pathologists, oncologists, and scientists collaboratively translate findings into actionable decisions [82]. Broader adoption will require standardisation, assay validation, harmonised reporting frameworks, and integration with digital pathology.

Furthermore, beyond technical and clinical challenges, large-scale molecular profiling raises important ethical, regulatory, and data governance considerations. Comprehensive multiomics analyses may generate incidental or germline findings, requiring clear frameworks for informed consent, data disclosure, and patient counselling. In addition, the collection, storage, and sharing of high-dimensional molecular data pose challenges related to data privacy, security, and regulatory oversight, particularly across international consortia. Data integration remains challenging due to differences in platforms, data scales, and bioinformatic pipelines, while variability in sample processing and sequencing depth complicates reproducibility. Cost, infrastructure requirements, and turnaround times restrict comprehensive profiling to specialised centres, making these approaches less accessible in low-resource settings. Addressing these issues will require harmonised governance frameworks and alignment with evolving ethical and regulatory standards to ensure responsible implementation of integrated diagnostics in precision oncology.

## Figures and Tables

**Table 1:** Multiomics-guided therapeutic stratification in precision oncology.

Diagnostic Domain	Representative Assays	Clinical Interpretation	Impact on Treatment Decisions
Histopathology	Routine histopathologic examination using haematoxylin–eosin staining	Tumour classification, differentiation, invasion patterns	Establishes baseline diagnosis and informs site-specific therapy
Immunophenotypic profiling	Single-plex and multiplex immunohistochemical assays	Target and immune marker expression (HER2, PD-L1), tumour microenvironment	Determines eligibility for targeted and immunotherapies
Genomic profiling	Clinically validated somatic mutation and copy number testing	Oncogenic drivers, resistance alterations, genomic instability	Guides use of approved targeted therapies and clinical trial options
Transcriptomic profiling	Gene expression-based classifiers and immune signatures	Molecular subtypes and pathway activation status	Supports prognostic stratification and treatment selection
Proteomic and phosphoproteomic profiling	Quantitative protein and signalling pathway activity assessment	Functional pathway dependence and post-translational regulation	Informs drug prioritisation and combination strategies
Metabolomic profiling	Tumour and biofluid metabolic profiling	Metabolic reprogramming and adaptive resistance mechanisms	Identifies potential metabolic targets and resistance mechanisms
Computational integration	Integrated bioinformatic and decision support framework	Cross-domain pathway convergence and therapeutic dependencies	Refines therapeutic ranking and combination strategies
Clinical interpretation	Multidisciplinary molecular tumour boards	Integration of morphologic, molecular, and clinical data	Enables personalised treatment planning and trial matching

## Data Availability

No new data were created or analysed in this study. Data sharing is not applicable to this article.
